# Morphological and Functional Principles Governing the Plasticity Reserve in the Cerebellum: The Cortico-Deep Cerebellar Nuclei Loop Model

**DOI:** 10.3390/biology12111435

**Published:** 2023-11-16

**Authors:** Hiroshi Mitoma, Shinji Kakei, Hirokazu Tanaka, Mario Manto

**Affiliations:** 1 Department of Medical Education, Tokyo Medical University, Tokyo 160-0023, Japan; 2 Department of Anatomy and Physiology, Jissen Women’s University, Tokyo 191-8510, Japan; kakei-shinji@jissen.ac.jp; 3 Faculty of Information Technology, Tokyo City University, Tokyo 158-8557, Japan; hirokazu@jaist.ac.jp; 4 Cerebellar Ataxias Unit, Department of Neurology, Médiathèque Jean Jacquy, CHU-Charleroi, 6042 Charleroi, Belgium; mario.manto@ulb.be; 5 Service des Neurosciences, University of Mons, 7000 Mons, Belgium

**Keywords:** cerebellar reserve, cerebellar ataxias, internal forward model, predictive control, synaptic plasticity, long-term depression

## Abstract

**Simple Summary:**

We propose a more comprehensive scheme underlying cerebellar reserve. Under pathological conditions, the internal forward model continuously updates itself to adjust the predictive control, where two reorganizing steps function cooperatively: updating predictions in the residual or affected cerebellar cortex (predictive step) and adjusting the updated predictions with the current status at the cerebellar nuclei (filtering step).

**Abstract:**

Cerebellar reserve compensates for and restores functions lost through cerebellar damage. This is a fundamental property of cerebellar circuitry. Clinical studies suggest (1) the involvement of synaptic plasticity in the cerebellar cortex for functional compensation and restoration, and (2) that the integrity of the cerebellar reserve requires the survival and functioning of cerebellar nuclei. On the other hand, recent physiological studies have shown that the internal forward model, embedded within the cerebellum, controls motor accuracy in a predictive fashion, and that maintaining predictive control to achieve accurate motion ultimately promotes learning and compensatory processes. Furthermore, within the proposed framework of the Kalman filter, the current status is transformed into a predictive state in the cerebellar cortex (prediction step), whereas the predictive state and sensory feedback from the periphery are integrated into a filtered state at the cerebellar nuclei (filtering step). Based on the abovementioned clinical and physiological studies, we propose that the cerebellar reserve consists of two elementary mechanisms which are critical for cerebellar functions: the first is involved in updating predictions in the residual or affected cerebellar cortex, while the second acts by adjusting its updated forecasts with the current status in the cerebellar nuclei. Cerebellar cortical lesions would impair predictive behavior, whereas cerebellar nuclear lesions would impact on adjustments of neuronal commands. We postulate that the multiple forms of distributed plasticity at the cerebellar cortex and cerebellar nuclei are the neuronal events which allow the cerebellar reserve to operate in vivo. This cortico-deep cerebellar nuclei loop model attributes two complementary functions as the underpinnings behind cerebellar reserve.

## 1. Introduction

**Figure 1:**
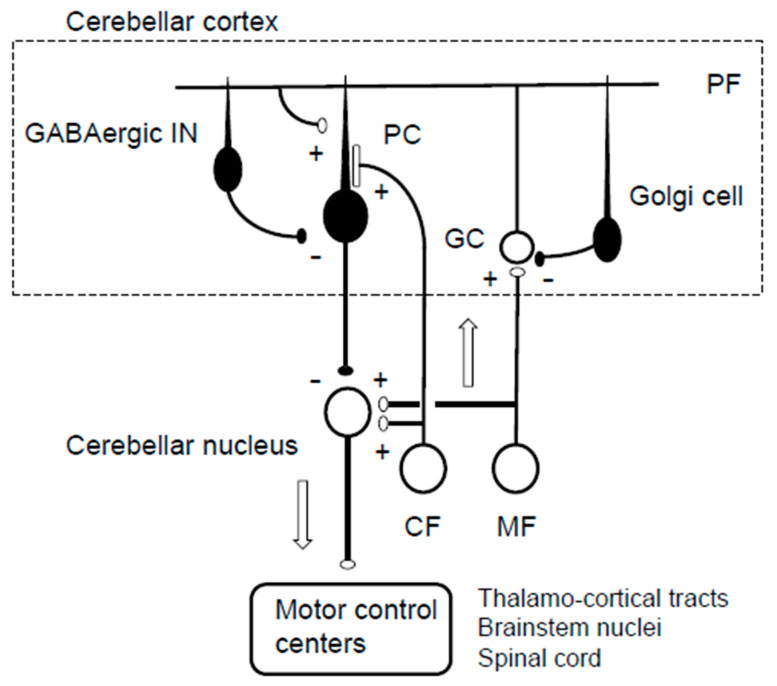
A scheme of the cerebellar neural circuit. MF, mossy fiber; CF, climbing fiber; GC, granule cell; PF, parallel fiber; PCs, Purkinje cells; GABAergic IN, stellate and basket cells; + excitation [open circles, excitatory neurons]; − inhibitory [filled circles, inhibitory neurons].

Cerebellar circuitry is unique due to its geometrical cytoarchitecture, its neuronal composition, and its connectivity. Figure 1 illustrates the homogeneous neural circuit in the cerebellar cortex. The intrinsic neuronal connections in the cerebellar cortex were first described in the 1960s by Eccles and colleagues [1]. The cerebellar cortex receives two excitatory inputs: mossy fibers (MFs) and climbing fibers (CFs) [1,2]. MFs convey information from the periphery and the cerebral cortex and make synaptic connections in the granule cells (GCs). The parallel fibers (PFs), axons of the GCs, make en-passant-type excitatory synapses on the distal dendrites of Purkinje cells (PCs) arranged in a mediolateral direction [1,2]. Individual PCs receive inputs from more than 100,000 PFs [3]. In comparison, a single CF originating from the inferior olive nucleus (IO) makes multiple synapses on the proximal dendrites of PCs arranged in a rostrocaudal direction [1,2]. The inhibitory interneurons in the cerebellar cortex exhibit two types of inhibitory modulation: Golgi cells driven by PF inputs make inhibitory synapses on GCs, whereas stellate cells and Basket cells, also driven by PF inputs, inhibit PCs to offset PF-induced PC excitement [1,2]. PCs, the sole output cells from the cerebellar cortex, integrate the PF- and CF-mediated excitatory signals and the inhibitory interneuron-mediated inhibitory signals and send their inhibitory signals to the cerebellar nucleus neurons [1,2].

**Figure 2:**
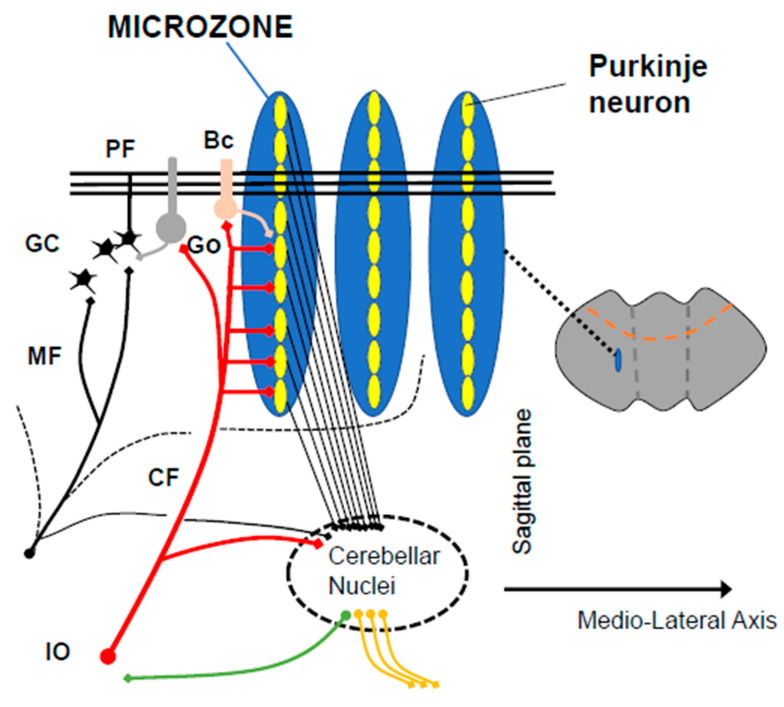
A scheme of microzones. Functional congruence between the 2 major input systems (mossy fibers, climbing fibers) is observed anatomically, with a contribution of mossy fibers into multizonal microcomplexes integrated in cerebellar modules subserving the operational aspects of the cerebellar machinery. PF: parallel fiber, CF: climbing fiber, GC: granule cell, Go: Golgi cell, Bc: basket cell, IO: inferior olive. Citation from our previous paper [5], with permission.

Based on further anatomical research on the input–output organization of the cerebellar cortex, the cerebellar cortex is currently assumed to have a slit-shaped functional unit, termed the microzone, which spreads in a rostrocaudal direction [4] (Figure 2). In the microzone, PCs receive CF inputs from a restricted region in the IO and, in turn, send their inhibitory outputs to neurons in a limited area of the cerebellar nuclei (CN) [4].

It is not surprising that the unique structure of the cerebellum, often described as having a crystal-like architecture, has attracted the interest of many researchers and promoted research on the learning function of the cerebellum. The theory advocated by Marr–Albus–Ito [2] assumed that long-term depression (LTD) at PF-PC synapses serves as a supervised learning machinery. CF inputs that convey error signals trigger a complex spike in PCs, which induces LTD on the PF-PC synapses [2]. Thus, according to the Marr–Albus–Ito model, the output signals from the cerebellar cortex are optimized by adjusting the PF-PC synapses using the error (teacher) signals fed back via the CFs. Since the 1980s, this theory has been supported by the results of various behavioral experiments, such as vestibular–ocular reflex or eye blink conditioning in animals treated with pharmacological agents and genetically engineered signaling cascades [6].

However, various aspects of the Marr–Albus–Ito theory have been challenged. First, based on the presence of multiple forms of synaptic plasticity in the cerebellar cortex, some researchers argue that the learning process is not limited to the PF-PC synapses but represents the orchestration of plasticity at multiple synaptic sites [7]. Second, the results are in line with the hypothesis that CFs convey an error signal [8]. Finally, others argue that since the cerebellum is involved in cognitive controls [9], where the learning process might rely on reinforcement learning rather than supervised learning [10], maximizing the reward will be more critical than having desired outputs [10]. Thus, a framework that unifies the mechanisms of motor and cognitive learning in the cerebellum has yet to be theorized [10].

On the other hand, the unique clinical feature of spontaneous/autonomous improvement in cerebellar symptoms following an injury may provide a new perspective on the theory of cerebellar plasticity and learning. Various pathologies can result in cell destruction in the cerebellum, but surprisingly, unlike other parts of the central nervous system, the cerebellum can self-correct the altered functions per se, resulting in an improvement in cerebellum-related clinical features. This remarkable phenomenon was first described in Holmes’ classic paper (pages 514–515) [11]. This outstanding capacity for compensation and restoration following pathological injury is defined as cerebellar reserve [5,12,13,14,15]. To date, two types of cerebellar reserve have been described [14]. The first, the structural cerebellar reserve, refers to a cerebellar area that compensates for impairment associated with damage to another localized cerebellar area induced by acute and transient pathology, such as vascular disease or trauma [14]. The second, termed the functional cerebellar reserve, refers to a cerebellar area that resists pathological impairment, through functional or structural reorganization, following gradual and progressive degeneration of a localized cerebellar region, such as that seen in immune-mediated diseases (subacute time-course) and degenerative or metabolic diseases (chronic and progressive time-course) [14]. Although plasticity is assumed, the exact mechanism of how cerebellar function is compensated and maintained is currently unknown.

Clarification of the mechanisms of cerebellar compensation has a major impact on our appraisal of brain function and attempts to promote recovery following brain injury in general. We propose, in this paper, a novel mechanism for understanding the cerebellar reserve at the neural circuitry level and thence apply such a mechanism to enhance our understanding of cerebellar plasticity. Toward this goal, we first review recent clinical studies that have documented the structural and functional features of the cerebellar reserve. Clinical data indicate that the collaborative function of the cerebellar cortex and cerebellar nuclei underlies cerebellar reserve. Second, we propose that the Kalman filter, which consists of a prediction stage in the cerebellar cortex and a filtering stage in the cerebellar nuclei [16], operates not only as a predictive controller but also as a neural device that promotes plasticity. Finally, based on these clinical and physiological data, we discuss the possible neural mechanisms underlying cerebellar reserve. We propose a scheme that integrates two elemental functions at the cerebellar cortex and cerebellar nuclei.

## 2. Recovery Process and Structural Plasticity in Cerebellar Pathologies

In order to examine the clinical pathophysiology underlying structural and functional cerebellar reserve, we examined the recovery process in acute etiologies and the structural plasticity in imaging studies with chronic etiologies. We performed a PubMed search using the keywords/terms [(“cerebellar stroke” AND “recovery” OR “reversibility” OR “compensation”) AND “2009–2023”] (31 results) and [“functional MRI” OR “voxel-based morphometry” AND “cerebellar degeneration” AND “2009–2022”] (329 results). We also examined how synaptic plasticity is impaired in animal models of spinocerebellar ataxia using a PubMed search using the keywords/terms [(“spinocerebellar ataxia” AND “LTD” AND “2009–2023”] (60 results). We read the abstracts of these articles and focused on eight relevant articles [17,18,19,20,21,22,23,24].

### 2.1. Reversibility after Acute and Transient Pathology

Several recent studies have provided descriptions of the recovery processes and range of compensatory lesions. We (M.M.) showed that compensation following cerebellar stroke is a step-by-step process [25]. The recovery process of hypermetria in goal-directed wrist movements is classified into four stages: hypermetria that does not change with weight bearing (stage 1), hypermetria exacerbated by weight bearing (stage 2), hypermetria that appears under weight bearing (stage 3), and complete recovery (stage 4). Stage 2 corresponds to the period when the agonist muscle EMG can be increased during weight bearing. Stage 3 corresponds to the period when the delayed latency of the antagonist muscle can be improved. Finally, stage 4 corresponds to the period when the antagonist muscle EMG can be increased during weight bearing. On the other hand, another study reported maladaptation after cerebellar stroke. The authors examined recovery using subjective visual vertical (SVV) after cerebellar stroke (lobule V, VI, and VIIa) [18]. Five of the twenty-two study patients showed pathological deviations of SVV in the acute phase. Notably, four of five patients showed shifts of SVV to the opposite side, suggesting overcompensation. These studies highlight that compensation is a complex phenomenon with multiple facets.

Studies examining the correlation between the spread of acute and transient lesions and the reversibility of symptoms offer implications for mechanisms of plasticity. Konczak and colleagues described the recovery processes of upper limb function after cerebellar stroke in 16 patients using methods of detailed lesion mapping and arm kinematics [17]. During the acute stage, these patients developed slowness of velocity and acceleration in hand movements [17]. At two-week follow-up, the slowness improved dramatically, and, for example, the gain in acceleration improved by up to 86% [17]. Notably, lesions of the cerebellar cortex and cerebellar nuclei (the dentate and interpositus nuclei) were associated with persistent problems in arm control [17]. The same group also examined the recovery from brain tumor resection in 22 children and adolescents and reported that lesions affecting the deep cerebellar nuclei were not fully compensated for at any developmental age [26].

The above clinical studies suggest that recovery from acute and transient pathologies is a step-by-step process and can sometimes be excessive, and that such structural cerebellar reserve requires the survival and proper functioning of the cerebellar nuclei.

### 2.2. Plasticity during Chronic and Progressive Pathologies

The benefits of motor rehabilitation in patients with degenerative cerebellar ataxia (CA) are now well established. Ilg and colleagues showed that a four-week intensive training course improved CAs in patients with degenerative CA, and such benefits were apparent for up to one year [27]. These studies suggest that resilience to progression is maintained even in degenerative diseases.

***Synaptic plasticity in degenerative disease***. Deficits in cerebellar synaptic plasticity have been reported in model mice with spinocerebellar ataxia (SCA), along with impaired LTD at PF-PC synapses in SCA14 model mice [19], impaired LTP and LTD at PF-PC synapses in SCA6 model mice [20], and impaired LTD at PF-PC synapses in SCA1 model mice [21]. These malfunctions of synaptic plasticity may be secondary to weakened synaptic function by cellular degeneration. However, an alternative mechanism is the dysfunction of a molecule key to the induction of synaptic plasticity: PKCγ in SCA14, P/Q-type Ca^2+^ channel in SCA6, and metabotropic glutamate receptor type 1 in SCA1 [19,20,21]. In one study using SCA1 model mice, the modulation of mGluR1 led to a reinforcement of LTD and an improvement in ataxic symptoms [28]. Thus, pathological conditions associated with LTD disorders have a characteristic feature of exacerbation of ataxia due to a lack of compensation. Accordingly, we like to consider them under the spectrum of “LTDpathy” [29].

In conclusion, animal model studies show an impairment in synaptic plasticity in certain degenerative diseases and show that its restoration improves CAs, suggesting that synaptic plasticity plays a key role in relieving CAs and serves as the resilience to symptom manifestation.

***Plasticity in cerebellar efferent pathways***. In Lurcher mice, cerebellar cortical degeneration is followed by increased efficacy of neurons of various cerebellar nuclei [30]. In this regard, a study of an animal model of alcohol-toxicity-associated CA showed training-related fine structural remodeling in the cerebellum [31]. Motor training improved ataxic symptoms in rats, showing synaptogenesis of remaining PCs and an increase in the size of astrocytes [31].

Human imaging studies have identified natural-course-related or training-related volume changes in the cerebellum and cerebrum of patients with degenerative CA. For example, Draganova and colleagues reported complementary volume changes in MRI with the progression of degenerative pathology in 30 patients with degenerative CA [22]. The gray matter volume decreased in the cerebellar cortex, mainly in lobules IV, V, VI, VIII, Crus I, and Vermis [22]. In comparison, an increase in the gray matter volumes of the dentate nucleus (DN), premotor cortex, and supplementary motor area was noted [22]. On the other hand, Burciu and colleagues examined the effects of two-week postural training on gray matter volume using voxel-based morphometry in 19 patients with degenerative CA and age-matched healthy subjects [23]. The two-week balance training improved the stability in the healthy subjects and patients with degenerative CA. Notably, the increase in gray matter volume following the balance training was reversed in patients with degenerative CA and the control subjects. Interestingly, the increase in gray matter volume of the dorsal premotor cortex was more prominent in the patients than in the controls, whereas the increase within the cerebellum, lobule VI, and Crus I was more prominent in the control subjects than in the patients. A similar volume increase in the premotor cortex was reported in another study that examined the effects of visuomotor training in 40 patients with degenerative CA [24]. The observed increase in gray matter volume was assumed to be due to neurogenesis, synaptogenesis, changes in neuronal morphology, and extra-neuronal changes (increases in glial cell size and number and angiogenesis) [32].

The results of these clinical studies suggest that the degenerative process affecting neurons of the cerebellar cortex causes a decrease in gray matter volume in the cerebellar cortex, and also induces structural plasticity in the form of increased gray matter volume in the cerebellar nuclei, the supplementary motor area, and the premotor cortex, i.e., the cerebellar efferent pathway (the dentate–thalamo–cerebral pathway). In other words, such structural plasticity in the cerebellar efferent pathway is a component of the functional cerebellar reserve in chronic and progressive pathologies.

### 2.3. Summary of Clinical Studies

Based on the above review, clinical studies suggest that the following principles underlie structural and functional cerebellar reserve:

***Induction of plasticity***. Synaptic plasticity in the cerebellar cortex may be involved in reversing symptoms of acute and transient pathologies and arresting the progression of chronic and progressive pathologies.

***Conditions for plasticity***. The cerebellar nuclei, or the cerebellar efferent pathways, are essential for recovery and resilience. Morphological changes (e.g., thickening of the efferent pathways) are known to accompany chronic and progressive pathologies. In this regard, clinical data indicate that any theory that can explain the concept of cerebellar reserve requires a framework that defines the cooperative work of the cerebellar cortex with cerebellar nuclei.

***Step-by-step process and malfunctioning of plasticity***. The recovery process from acute and transient pathologies shows a step-by-step pattern, although it can also sometimes show an intermittent/abrupt pattern. The direction of the step-by-step recovery process may proceed either toward recovery or toward maladaptation (aberrant recovery, overcompensation).

## 3. Predictive Controller Generating Cerebellar Reserve

**Figure 3:**
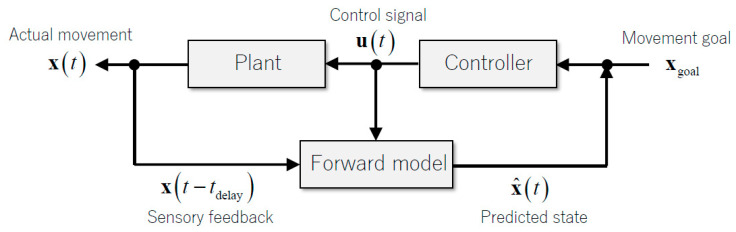
Computational diagrams of internal models. The internal forward model integrates sensory feedback signals and the efference copy of a control signal and predicts the current status of the body. Citation from our previous paper [39].

An internal forward model transforms a motor command into a prediction of its outcome in terms of the sensory reafference that the movement will cause [33,34,35] (Figure 3). Accumulating evidence suggests that the internal forward model embedded in the cerebellum controls motor accuracy online [16,36]. On the other hand, computational studies have shown that motor adaptation or learning is achieved by changes in the internal models within the nervous system, and perhaps specifically within the cerebellum [34,37].

There is cross talk between the online control and learning process. Motor learning compares the difference between the actual and predicted outcomes of a motor action to reduce this difference [38]. Physiological studies have shown that sensory prediction errors drive reaching adaptation during a visuomotor task [37]. Thus, the predictive control drives motor learning toward further improvement [38]. In other words, continuing predictive control to achieve accurate motion ultimately promotes the learning processes.

These studies suggest the reinstitution of the cerebellar predictive controller in the event of injury, and that the loss of function gradually leads to recovery while repeating the predictive control. Here, we provide an outline of the neural substrate of the cerebellar predictive controller and discuss the mechanisms required for updating predictive control in order to clarify the physiological mechanisms underlying cerebellar reserve.

### 3.1. Internal Forward Model and Kalman Filter

Sensory feedback signals have a delay of several tens to one hundred milliseconds to travel from peripheral sensory organs to the sensory areas in the brain. Therefore, when a feedback controller depends on the delayed sensory feedback for motor control, the movement outcomes become unsteady and jerky, with oscillations [16,35]. Recent computational studies have assumed that the cerebellum functions as an internal forward model controller to coordinate various movements, i.e., a controller that predicts the future state of a motion consequence by solving dynamics with a given efference copy of motor commands and the current states [16,35,36]. This assumption relies on the anatomical features of the cerebellar input–output organization. The cerebro-cerebellum, forming a closed-loop connection between cortical motor areas, receives both the efference copy and somatosensory inputs required for a neuronal substrate to serve as a forward model [16]. In our previous study (studies by S.K. and H.T.) that analyzed neural activities in monkeys performing step-tracking movements [39], the firing rate of mossy fibers (MFs) at time *t* + *t*_1_ (the neural activity encoding a future state) was well reconstructed as a weighted sum of dentate nucleus neurons at time *t* (the neural activity representing the current output of the cerebellum). This analysis provides direct neural evidence for the internal forward model hypothesis of the cerebellum.

**Figure 4:**
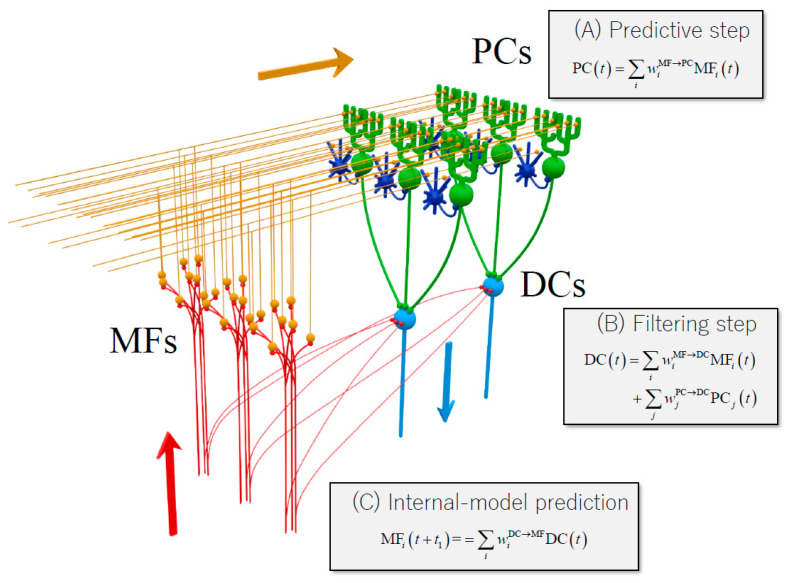
Summary schematic of the internal forward model in the cerebellar cortex. MF, mossy fiber (red); PC, Purkinje cell (green); DC, dentate cell (light blue). Granule cells (orange) and inhibitory interneurons (blue). The linear equations of neuron activities resemble those of an estimator known as the Kalman filter. Citation from our previous paper [39].

In addition, a set of linear equations have been created to describe the firing rates between cerebellar neurons [39]. First, the firing rate of PC was constructed linearly as the weighted sum of the firing rate of MF. Second, the firing rate of cells of the dentate nucleus (DNCs) was constructed linearly as the weighted sum of the firing rates of PC and MF. These linear equations matched those of the optimal predictor known as the Kalman filter [39]. In the Kalman filter framework, the predictive step computes the predictive state (PC) from the current state (MF), and the filtering step (DC) integrates the predictive state (PC) and sensory feedback from the periphery (MF) (Figure 4) [39]. Here, we postulate that MF projecting to PC represents the current estimate of the state and the motor efference copy, and that MF with collaterals to DC provides sensory feedback. Therefore, the process of compensation and restoration for cerebellar lesions must restore the following two functions: updating predictions in the residual or affected cerebellar cortex and adjusting its updated predictions with the current status at the cerebellar nucleus. In the following Section 3.2 and Section 3.3, we discuss the possible neural mechanisms underlying plasticity in the cerebellar cortex and cerebellar nuclei.

### 3.2. Reorganization of the Cerebellar Cortex for Predictive Behavior

Pathological damage of the cerebellar cortex is followed by reorganization of the prediction step, either at a site unaffected by the pathological process or at the site of damage/impairment. The mechanisms underlying the restructuring of predictive computation could include a specific morphological and functional mechanism of “redundancy” and “multiple forms of synaptic plasticity”.

***Redundancy***. About 60–80% of the 85–100 billion neurons of the brain are located in the cerebellum, although the cerebellum only forms about 10% of the brain mass [40,41]. Furthermore, the GCs form the largest bulk of the cerebellar cells, totaling approximately 50 billion cells [42]. Consequently, the cerebellar circuit undergoes a process of expansion and compression from input to output connectivity, i.e., expansion from MF (the cerebellar inputs) to GC (with a ratio of 2:1600, normalized under the condition that the number of PCs is 1.0) and compression from GC to PC (with a ratio of 1600:1) and to the cerebellar nuclei (the cerebellar outputs) (with a ratio of 1600:0.038) [16].

**Figure 5:**
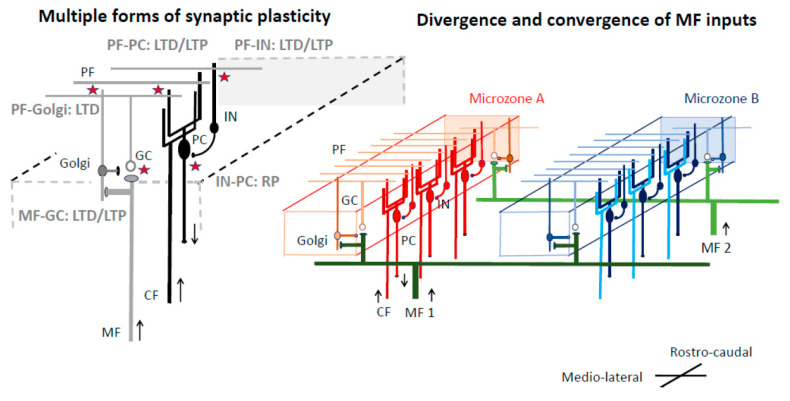
A schematic diagram of circuits within the cerebellar cortex, cited from our previous paper [13]. PC: Purkinje cell, GC: granule cell, INs: molecular layer interneurons; Golgi: Golgi cell; MF: mossy fiber, PF: parallel fiber, CF: climbing fiber. Two cellular mechanisms underlying cerebellar reserve are illustrated. (*1*) Multiple forms of synaptic plasticity (illustrated by stars) co-exist in the cerebellar cortex. (*2*) Convergence and divergence of MF inputs. For example, MF1 innervates both microzones A and B. Different MFs converge simultaneously to multiple microzones. Thus, a single microzone receives abundant central and peripheral inputs through MF, which results in redundancy of information.

Another feature of the cerebellum is that cerebellar neurons reside in microzones, which represent virtual functional units that extend along the rostrocaudal axis [4,43]. The PCs in a microzone receive inputs from the CF, which are derived from cells localized in IO and, in turn, are projected to localized sites in CN [4,43]. Notably, the axons of a single MF extend in a mediolateral direction perpendicular to the direction of microzones, thus projecting to multiple microzones [44,45] (Figure 5). Consequently, a single microzone receives information from many MFs that carry signals from the cerebral cortex and periphery. In support of this peculiar organization, recent physiological studies have shown that a sizable number of GCs increase their activities during learning tasks [46], and individual GCs receive convergent functional multimodal (somatosensory, auditory, and visual) inputs via separate MFs [47]. Another recent study argued that only a few GCs are active at a time [48], suggesting that although the majority of GCs are not activated under a particular condition, they do receive particular inputs about the condition and have the potential to be activated after the reorganization.

In conclusion, two conditions are required to update the prediction step in the cerebellar cortex: (1) the survival of a sufficient number of cells in the spared area and (2) the availability of MF input to convey the current status. “Redundancy”, satisfying these two conditions, can be seen in the enormous number of GCs and the diffuse and extensive projection pattern of MF.

***Multiple forms of synaptic plasticity***. Multiple forms of synaptic plasticity operate cooperatively in the cerebellum [7,49] (Figure 5), including spike-timing-dependent plasticity at MF-GC synapses [50], LTD at PF-PC synapses [51,52], LTP at PF-PC synapses [53,54], rebound potentiation of inhibitory inputs to PC [55], aLTP/ LTD at PF-stellate cell synapses [56], and plasticity at MF-CN cell synapses [57].

Cerebellar synaptic plasticity has been implicated in adaptative tasks, such as vestibular–ocular reflex or eye blink conditioning [58]. On the other hand, studies using animal models have also shown the involvement of synaptic plasticity in the compensatory function under pathological conditions affecting the cerebellum [59], and that an enhancement in synaptic plasticity is associated with an improvement in ataxic symptoms [28]. Furthermore, recent clinical studies have attempted to reinforce synaptic plasticity using non-invasive cerebellar stimulation to compensate for lost functions in various cerebellar disorders [15,60,61].

The modification of synaptic transmission could potentially compensate for or repair impaired predictive computation from an MF-conveyed current state to a PC-conveyed predictive state in the cerebellar cortex. Synaptic plasticity might reproduce the optimal conditions for the predictive step at substitution sites (structural cerebellar reserve), whereas it might enhance attenuated transmissions at injury sites (functional cerebellar reserve). Either error-based or reward-based predictive computation may promote synaptic-plasticity-mediated reorganization [38]. Therefore, “multiple forms of synaptic plasticity” would favor remodeling the predictive step in the cerebellar cortex.

### 3.3. Adjustment at Cerebellar Nuclei for Filtering Step

The cerebellum includes four nuclei: the fastigial, anterior and posterior interpositus, and dentate nuclei. DNCs receive inputs from PC in the cerebro-cerebellum. The DNC sends collaterals to the red nucleus (RN) from the dentatofugal tracts reaching the thalamus [62]. The dentato–thalamo–cerebral pathways send outputs mainly back to the same area of the cerebral cortex, from which the cerebellum receives inputs [63].

An impairment in DNC function has historically been discussed within the framework of the Guillain–Mollaret (G–M) triangle [64]. The G-M triangle is marked within the DN, RN, and IO, and the disruption of each pathway causes tremor or tremor-like movements [64]. In harmaline-induced tremor model rodents, a large number of IO neurons appear to be discharged in synchrony and rhythmically, suggesting that IO plays a critical role in the generation of tremors [65]. On the other hand, we argue that an impairment in DNC function should not only be considered within the framework of tremor generation but also within the framework of dysplasia of the cerebellar reserve. Physiological and computational studies indicate that DNCs adjust PC-mediated predictive signals with the MF-mediated current states (filtering step), which is in agreement with the clinical finding of a lack of recovery of ataxic symptoms following acute and transient lesions of cerebellar nuclei, resulting in failure of the cerebellar reserve.

The reasons for the hypertrophy of cerebellar nuclei and their target cerebral cortex observed in chronic cerebellar cortical disorders remain unclear. Two physiological findings may provide relevant insights. First, DNCs can regenerate after the decussation of the superior cerebellar peduncle [66], suggesting the potential for structural plasticity. Second, a study using the horizontal optokinetic response of ocular movements showed the occurrence of short-term adaptation first in the cerebellar cortex, followed by long-term adaptation at the vestibular nucleus [67], suggesting a shift in transsynaptic plasticity from the cerebellar cortex to the cerebellar nuclei.

The results of these physiological studies suggest that the cerebellar nuclei are not merely relay nuclei for outputs from the cerebellar cortex, but rather they serve as a filter that is indispensable for cerebellar reserve.

## 4. Conclusions

Traditional learning or compensatory mechanisms have mainly been attributed to synaptic plasticity in the cerebellar cortex. We propose an original and more comprehensive scheme underlying cerebellar reserve. Under pathological conditions, the internal forward model is continuously updated so as to adjust the predictive control, where two reorganizing steps function cooperatively: updating predictions in the residual or affected cerebellar cortex (predictive step) and adjusting the updated predictions with the current status at the cerebellar nuclei (filtering step) (Figure 4). There is consensus that constant revision of the internal forward model will ultimately lead to accelerated learning. Consistently, accumulating clinical data clearly show that when cerebellar nuclei are preserved, the cerebellar symptoms recover, whereas when the cerebellar nuclei are damaged together with the cortex, the cerebellar symptoms do not recover and become permanent, impacting on daily life.

In line with this assumption, further studies need to clarify the following questions. First, more systematic preclinical and clinical studies are needed to confirm that CAs can be improved by promoting or repairing synaptic plasticity in the predictive step in the cerebellar cortex. Second, it is necessary to clarify the mechanism of morphological plasticity of the cerebellar nuclei or the cerebellar efferent tract. In addition, the efferent signals from the DN feed back to the PC as CF input via RN and IO, and the functional meaning of this pathway in the cerebellar reserve needs to be experimentally clarified. Finally, it is necessary to clarify the relationship with existing cerebellar learning theories or reorganization theories such as cross-modal plasticity [68]. Cross-modal plasticity is an adaptive feature of the brain, whereby the loss of one sensory modality induces cortical reorganization that leads to enhanced sensory performance in remaining modalities.

Our proposal has implications for the management of cerebellar disorders mainly affecting the cerebellar cortex (such as some cases of immune-mediated ataxias targeting PCs, for instance) and those mainly affecting cerebellar nuclei (such as cerebellar tumors, for instance). In the first situation, therapies (drugs, noninvasive brain stimulation modulating PCs’ excitability, rehabilitation) aim to promote restoration of the predictive mechanisms. In the second situation, the filtering properties of nuclei have to be compensated. This second goal is highly challenging with the current techniques available and might explain why cerebellar patients with cerebellar nuclei lesions often exhibit long-lasting deficits, whereas patients with cerebellar cortical lesions tend to recover faster. Another implication is to better understand the implementation of the two elemental mechanisms as a function of the age of the patient [69].

Overall, a novel picture of cerebellar compensation is emerging. It is now clear that multiple forms of plasticity are required to settle the compensatory mechanisms. The model reported here brings a novel view in our appraisal of cerebellar function, underlining that the cerebellar cortex and cerebellar nuclei are two key structures whose activities allow for resilience when facing an injury. As compared to the Marr–Albus–Ito model, our model is oriented toward compensatory functions of cerebellar circuitry.

## Data Availability

Data is contained within the article.
